# Nursing Markedly Protects Postpartum Mice From Stroke: Associated Central and Peripheral Neuroimmune Changes and a Role for Oxytocin

**DOI:** 10.3389/fnins.2019.00609

**Published:** 2019-07-08

**Authors:** Creed M. Stary, Lijun Xu, Ludmilla A. Voloboueva, Marcela Alcántara-Hernández, Oiva J. Arvola, Juliana Idoyaga, Rona G. Giffard

**Affiliations:** ^1^Department of Anesthesiology, Perioperative and Pain Medicine, Stanford, CA, United States; ^2^Department of Microbiology and Immunology, Stanford, CA, United States; ^3^Program in Immunology, Stanford University School of Medicine, Stanford, CA, United States

**Keywords:** MCAO, FACS, ischemia, innate, adaptive, focal ischemia, inflammation, cytokine

## Abstract

Recent studies demonstrate significant neuroimmune changes in postpartum females, a period that also carries an increased risk of stroke. Oxytocin, a major hormone upregulated in the brains of nursing mothers, has been shown to both modulate neuroinflammation and protect against stroke. In the present study we assessed whether and how nursing modulates the neuroimmune response and injury after stroke. We observed that postpartum nursing mice were markedly protected from 1 h of transient middle cerebral artery occlusion (MCAO) relative to either non-pregnant/non-postpartum or non-nursing (pups removed) postpartum females. Nursing mice also expressed reduced levels of pro-inflammatory cytokines, had decreased migration of blood leukocytes into the brain following MCAO, and displayed peripheral neuroimmune changes characterized by increased spleen weight and increased fraction of spleen monocytes. Intranasal oxytocin treatment in non-pregnant females in part recapitulated the protective and anti-inflammatory effects associated with nursing. In summary, the results of the present study demonstrate that nursing in the postpartum period provides relative protection against transient ischemic stroke associated with decreased brain leukocytes and increased splenic monocytes. These effects appear to be regulated, at least in part, by oxytocin.

## Introduction

Over the past decades significant progress has been achieved in understanding the complex cellular and molecular mechanisms of stroke pathology. Various physiological conditions and interventions that modulate stroke outcome have been identified and extensively investigated (Lo et al., 2003; Lakhan et al., 2009; Moskowitz et al., 2010), but clinical translation has been elusive. While stroke is uncommon in young women, the peripartum period is associated with a significantly increased risk of stroke (Kittner et al., 1996; James et al., 2005). Notably, approximately 15% of pregnant women who experience a stroke die as a result, making it the 8th leading cause of pregnancy-associated deaths in the United States (Kochanek et al., 2013), and retrospective clinical studies demonstrate the risk of stroke remains significantly elevated in the postpartum period (Jaigobin and Silver, 2000; Kamel et al., 2014; Cheng et al., 2017). However, to date, essentially no research has been conducted on stroke in the postpartum period in rodent models.

Strong evidence supports the notion that modulation of the neuroimmune response critically influences lesion volume and overall outcome after ischemic stroke. While the healthy brain maintains an anti-inflammatory local milieu limiting destructive inflammation (Carson and Sutcliffe, 1999), stroke initiates both acute and long-lasting inflammatory processes characterized by release of pro-inflammatory molecules and infiltration of inflammatory cells into the ischemic brain (Stoll et al., 1998; Schwab et al., 2001; Iadecola and Anrather, 2011). Stroke-induced release of pro-inflammatory mediators and cytokines leads to brain cell damage and apoptosis (Choe et al., 2011; Vogelgesang et al., 2014), specifically tied to increased levels of pro-inflammatory cytokines IL-6 and TNF-α (Campbell et al., 1993; Rothwell and Relton, 1993; Meistrell et al., 1997; Lavine et al., 1998). Induction of stroke results not only in brain damage and local neuroinflammation, but also has profound effects on peripheral immune responses, promoting peripheral immune suppression, splenic atrophy and changes in circulating leukocytes (Offner et al., 2006; Lafargue et al., 2012).

Sex differences in outcome from stroke have long been appreciated (Sohrabji et al., 2017), with female animals generally being protected compared to male animals (Alkayed et al., 1998; Murphy et al., 2004). A recent review summarizes the current clinical evidence for sex differences in ischemic stroke, and highlights immune/inflammatory pathways that may contribute to these clinical differences (Chauhan et al., 2017). While pregnancy, parturition and lactation are major important physiological changes in females, these factors have been little studied in the context of stroke. The postpartum period induces significant changes in brain physiology and plasticity including altered neurogenesis (Darnaudery et al., 2007; Leuner et al., 2007), neuronal morphology and synaptic plasticity (Tomizawa et al., 2003; Haim et al., 2014), and astrocytic and oligodendrocytes function (Salmaso and Woodside, 2006; Maheu et al., 2009). Only recently have studies reported changes in maternal neuroimmune function during the postpartum period (Haim et al., 2014; Sherer et al., 2017). In particular, the postpartum period is associated with changes in pro- and anti-inflammatory cytokine levels (Haim et al., 2017). Oxytocin, a hormone produced in the paraventricular and supraoptic nuclei of the hypothalamus, is best known for its role in lactation and parturition. In addition, oxytocin signaling modulates social behaviors, feeding, and pain perception. It has been demonstrated that oxytocin administration provides neuroprotection after cerebral ischemia in male mice, preventing the increased injury seen with social isolation (Karelina et al., 2011). In addition, oxytocin treatment inhibited pro-inflammatory microglial activation both *in vivo* and *in vitro* (Yuan et al., 2016), and mitigated neuroinflammatory responses associated with maternal separation (Amini-Khoei et al., 2017).

In the present study we compared infarct volume and neurological function following transient middle cerebral artery occlusion (MCAO) in acute postpartum nursing female mice with age-matched, postpartum non-nursing (pups removed) mice and age-matched nulliparous female mice. We observed marked protection in nursing mice accompanied by decreased migration of leukocytes and reduced levels of brain pro-inflammatory cytokines in the brain, and increased numbers of leukocytes in the spleen. Because oxytocin is a major hormone upregulated during nursing, we also performed comparative studies between nursing, non-pregnant, and oxytocin-treated non-pregnant female mice following MCAO.

## Materials and Methods

### Animals

The study was conducted in accordance with National Institutes of Health guidelines for the use of experimental animals, and the protocols were approved by the Stanford Animal Care and Use Committee. Female 10 week old Swiss Webster mice (Charles River Laboratory, Wilmington, MA, United States) were used. All animals were housed in air-conditioned rooms in a controlled environment at 21 ± 2°C with seasonal lighting conditions (12 h of light and 12 h of darkness), with unrestricted food and water. Pregnant mice were allowed to deliver and nurse the newborn pups for 3 days before use in stroke studies. Control postpartum mice were deprived of all their pups after delivery and did not nurse for 3 days prior to use in studies. MCAO was performed in each experimental group immediately after the 3 day treatment period. Control non-pregnant female mice were also studied, with and without 3 days of oxytocin treatment. The total number of female mice used was 147 (MCAO 83, non-stroke 64).

### Intranasal Oxytocin Treatment

Oxytocin was administered intranasally for 3 days with a dose previously demonstrated to significantly increase brain oxytocin levels in male mice (Neumann et al., 2013). Briefly, synthetic oxytocin (Tocris Bristol, United Kingdom), 12 μl of a 1 μg/μl in saline solution, or control saline vehicle alone, was administered, the solution was applied alternately into each nares, using a pipette. The solution was allowed to diffuse into the squamous epithelium of both the left and right tunica mucosa nasi.

### Transient Focal Cerebral Ischemia

Focal cerebral ischemia was induced, and neuroscore and edema corrected infarct volume (percent of hemisphere) were assessed as described previously (Xu et al., 2015). In brief, mice were randomized to treatment groups. Under 1.5–2.0% isoflurane anesthesia, the common carotid artery was exposed and the external carotid artery ligated and cauterized. Unilateral MCAO occlusion was performed by inserting a 6-0 nylon monofilament surgical suture from Doccol Corporation (Sharon, MA, United States). The suture was secured, and the animal allowed to awaken. After 60 min, the animal was briefly re-anesthetized and reperfusion was initiated by filament withdrawal. Sham-operated mice were treated identically with the exception that no filament was inserted. Intraoperative rectal temperature was controlled in all animals between 36.5 and 37.5°C.

Neurological deficit score (Yang et al., 1994) was determined after 24 h reperfusion. Scores were (0) no deficit, (1) forelimb weakness, failure to extend forepaw; (2) torso turning to the ipsilateral side when held by tail, circling to affected side, (3) inability to bear weight on affected side, falling (4) no spontaneous locomotor activity. Any animal without a visible deficit, score of 0, was excluded from the study. The number of animals excluded from the study was four for control, three for oxytocin and three for the nursing group. Ischemic injury resulted in five deaths in the control group, and three deaths in each of the oxytocin and nursing groups.

### Brain Oxytocin and Cytokine Measurements

For *ex vivo* measurements of oxytocin and proinflammatory cytokines, mice were killed and perfused with 0.9% saline. The brains were removed and the peri-infarct areas and corresponding brain areas in sham control animals were isolated and immediately homogenized in cold phosphate buffered saline using a ratio of 1 g of tissue to 10 ml of reagent plus protease inhibitor mixture (G-Biosciences, St. Louis, MO, United States). The samples were centrifuged at 10,000 × *g* for 20 min at 4°C and the supernatants were used for measurements. Oxytocin levels in brain tissue were assayed by ELISA kit according to manufacturer’s instructions (DLdevelop Wuxi, China 214031). Levels of proinflammatory cytokines were determined by TNF-α and IL-6 ELISA kits (Invitrogen). Protein concentrations were measured by BCA protein assay (Pierce).

### Brain and Spleen FACS Studies

For FACS studies of brain immune cells, mice were euthanized and perfused with 20 mL of 0.9% saline. The brains were removed, chopped with dissecting scissors and digested with 400 mU/mL Collagenase-D (Roche, Germany) and 50 μg/mL DNase I (Roche) for 1 h in a 37°C incubator. 10 μM EDTA (Life Technologies, Grand Island, NY, United States) was added for the last 5 min. The samples were centrifuged at 450 × g for 10 min, resuspended in 4 mL of 67.5% Percoll (Sigma, St. Louis, MO, United States) and carefully overlaid with 4 mL of 30% Percoll. The Percoll gradient mix was centrifuged at 800 × g for 20 min at room temperature. The cells were collected at the Percoll interface (lymphocytes and monocytes) and bottom pellet (granulocytes).

Spleen cell suspensions were obtained by mechanical disruption and enzymatic digestion with 400 mU/ml Collagenase D and 50 μg/ml DNase I for 30 min at 37°C, and 10 μM EDTA was added for the last 5 min. Cell suspensions were lysed with ACK lysis buffer (Lonza, Walkersville, MD, United States) and filtered through a 70 μm filter. Total leukocyte count (CD45^+^) from spleen and brain were obtained using Countbright Beads (Thermo Fisher Scientific, Eugene, OR, United States) following manufacturer’s instructions. Cell suspensions were incubated 15 min at 4°C with CD16/CD32 (produced in house from 2.4G2 hybridoma, ATCC) to prevent binding of antibodies through Fc-receptor. Cell suspensions were then stained using the following antibodies obtained from eBiosciences, BD or Biolegend: F4/80 PerCPCy5.5 (BM8 clone), CD115 PE (clone AFS98), CD11c PE-Cy7 (clone N418), CD19 APC-A780 and APC-A700 (clone eBio1D3), CD3e APC-A780 (clone145-2C11), Ly6C efluor450 (clone HK1.4), MHCII A700 (clone M5/114.15.2), CD4 BUV395 GK1.5, Ly6G BUV395 (clone 1A8), CD8 BV510 (clone 53-6.7) and CD11b BV785 (clone M1/70). For lymphocyte quantification, cells were stained with the surface cocktail: CD49b FITC (clone DX5), CD25 PerCPCy5.5 (clone PC61), CD19 APC-A700 (clone 1D3), CD62L APC-A780 (clone MLE14), CD44 BV785 (clone IM7), CD4 BUV396 (clone GK1.5), TCRbeta eFluor450 (H57-597), and CD8 BV510 (clone 53-6.7). All surface stainings were performed at 4°C for 20 min. After surface staining, cell were fixed using FoxP3 transcription factor detection kit (eBiosciences) for at least 2 h. Cells were permebealized and stained with FoxP3 APC (clone FJK-16s) for 30 min at 4°C. Stained cells were acquired in a Fortessa X-20 and fcs files were analyzed using FlowJo.

### Statistical Analyses

Numbers of animals/group are indicated in figure legends. Data reported are means + SEM. Statistical difference was determined using *T*-test for comparison of two groups or ANOVA followed by Tukey correction for experiments with >2 groups using SigmaPlot (Systat Software, San Jose, CA, United States). *P* < 0.05 was considered significant.

## Results

**FIGURE 1:**
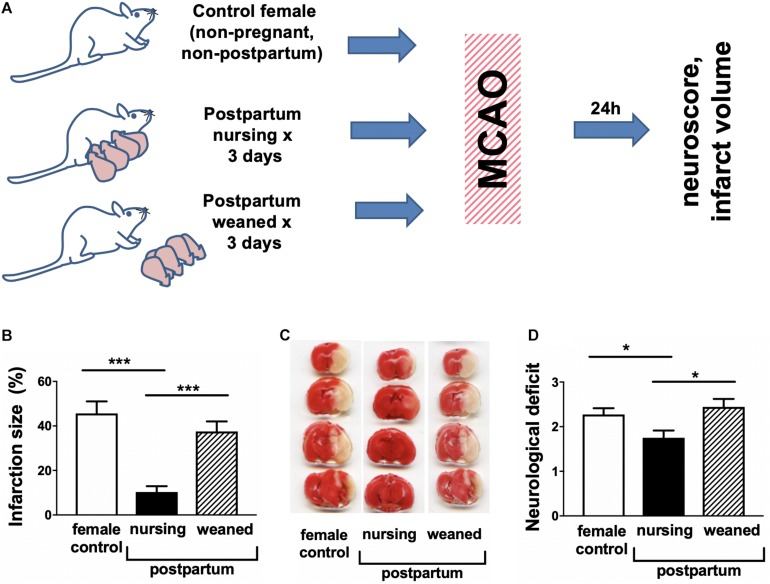
Nursing mice demonstrate marked protection from middle cerebral artery occlusion (MCAO). **(A)** Experimental overview. **(B)** representative TTC stained brain sections, **(C)** quantification of infarction volume as % of hemisphere, and **(D)** post-ischemic neurological deficit. Control are non-pregnant, age-matched females. (*N* = 8–10/group, ^∗∗∗^*p* < 0.001 and ^*^p < 0.05 compared to control).

Infarction volume was assessed by TTC staining 24 h after MCAO in non-pregnant/non-postpartum females, nursing postpartum females and postpartum females from which the pups had been removed after delivery (experimental overview illustrated in Figure 1A). We observed marked (>70%) reduction in nursing postpartum mice relative to either non-pregnant/non-postpartum mice or when nursing was inhibited by removing the pups in the postpartum period (Figures 1B,C). In parallel we evaluated changes in neurological score associated with nursing. Figure 1D demonstrates that nursing was associated with significantly improved neurological performance relative to non-pregnant/non-postpartum or non-nursing postpartum females. Because both stroke injury and neurobehavioral outcomes after MCAO were comparable between non-pregnant/non-postpartum mice and non-nursing postpartum mice, non-pregnant/non-postpartum mice were used as the “control” group in the remainder of studies.

**FIGURE 2:**
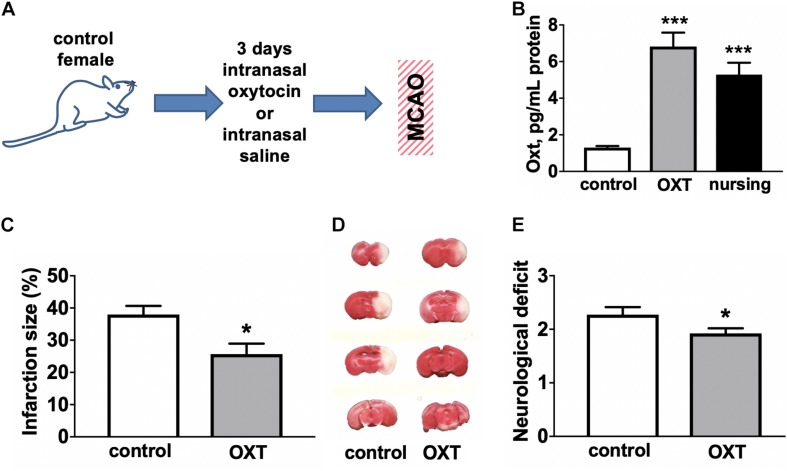
Oxytocin treatment improves post-MCAO outcome in female mice. **(A)** Experimental overview. **(B)** Intranasal oxytocin (Oxt) treatment for 3 days resulted in significantly higher brain levels of oxytocin than seen in non-nursing females and comparable to levels in nursing mice, *N* = 8–12. Oxytocin treated female mice demonstrated reduced infarction volume compared to control (Ctrl). **(C)** representative TTC stained brain sections, **(D)** quantification of percent of hemisphere infarct volume (*N* = 9–12), and **(E)** post-ischemic neurological deficit (*N* = 11–13) ^∗∗∗^*p* < 0.001 and ^*^p < 0.05 compared to control (Ctrl) non-nursing.

We next determined the effects of intranasally delivered oxytocin on stroke outcome (experimental overview illustrated in Figure 2A). We observed (Figure 2B) that 3d of intranasal oxytocin resulted in elevated brain oxytocin levels (5.2-fold) in control female mice relative to intranasal saline treatment, were comparable to levels observed in nursing females (4.1-fold). Administration of oxytocin resulted in a significant ∼(32%) decrease in the post-MCAO infarction volume (Figures 2C,D), significantly improved neurological scores (Figure 2E) compared to intranasal saline treated female controls.

**FIGURE 3:**
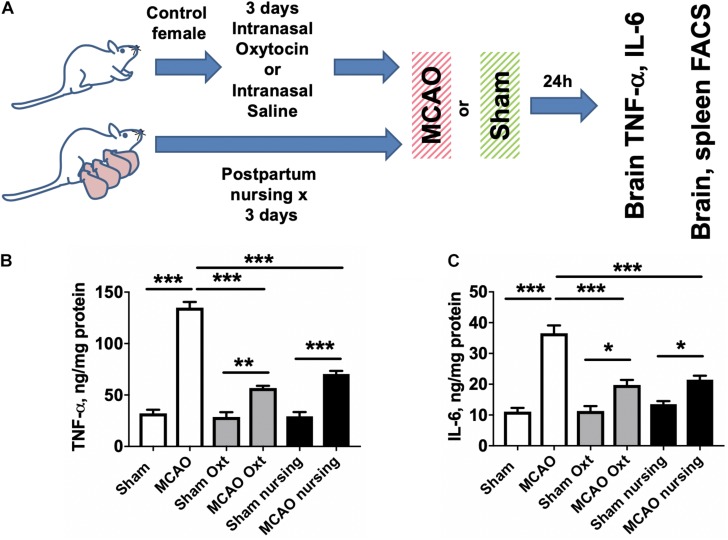
Increases in brain TNF- and IL-6 levels induced by MCAO are moderated by nursing or oxytocin treatment. **(A)** Experimental overview. Brain cytokine levels were assessed in sham operated or MCAO mice either nursing or treated with vehicle or oxytocin. TNF-α **(B)** and IL-6 **(C)** levels were significantly increased in mouse brain 24 h after MCAO compared to sham operated animals. This increase was significantly attenuated in both nursing and oxytocin (Oxt) treated mice *N* = 6 for MCAO animals, *N* = 4 for sham, ^∗∗∗^*p* < 0.001 ^∗∗^*p* < 0.01, and ^*^p < 0.05 for comparisons indicated by the horizontal bars.

Since it has been reported that both lactation and oxytocin treatment are associated with significant neuroimmunological changes (Yuan et al., 2016; Haim et al., 2017; Sherer et al., 2017), we measured brain levels of two major cytokines involved in post-ischemic brain injury, IL-6 and TNF-α, in non-pregnant/non-postpartum mice with and without intranasal oxytocin treatment and in nursing postpartum mice after MCAO (experimental overview illustrated in Figure 3A). We observed that brain TNF-α levels were significantly (4.2-fold) increased after MCAO in saline-treated control females, and this increase was significantly attenuated in both nursing postpartum and oxytocin-treated control females (Figure 3B). Similarly, brain IL-6 levels were significantly (3.3-fold) increased after MCAO in saline-treated control females, and this increase was also significantly attenuated in both nursing postpartum, and oxytocin-treated control females (Figure 3C). We did not observe any significant differences in brain TNF-α or IL-6 levels between the three groups in the absence of ischemic injury (sham surgery).

**FIGURE 4:**
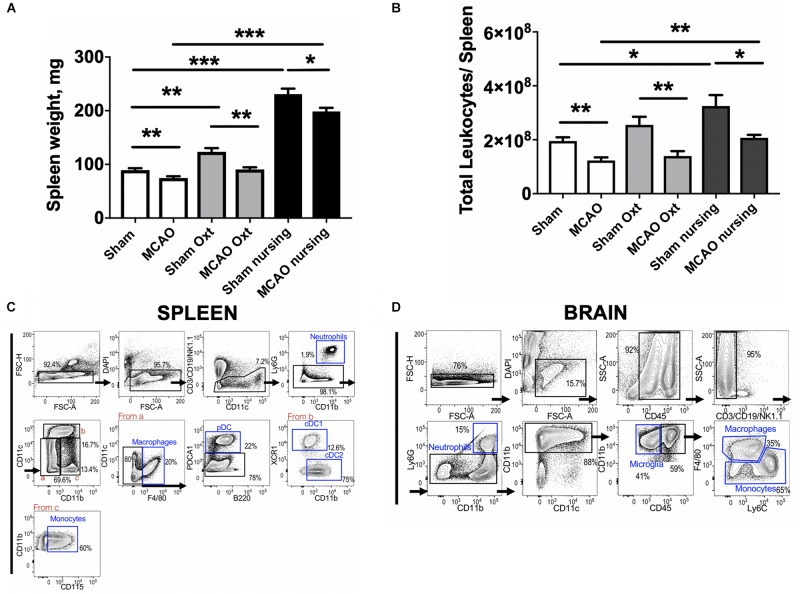
Changes in spleen 24 h past ischemia in nursing and oxytocin treated mice evaluated by spleen weight and FACS studies of leukocytes. Oxytocin (Oxt) treated and nursing animals demonstrated significantly bigger spleen weights **(A)**. Changes in total leukocyte numbers promoted by nursing and oxytocin treatment **(B)**. Sequential gating strategy to analyze myeloid cells in the spleen **(C)** and the brain **(D)**. Number represent frequency of parent. *N* = 11 for A, *N* = 4 for B, ^∗∗∗^*p* < 0.001, ^∗∗^*p* < 0.01, and ^*^p < 0.05, for comparisons indicated by horizontal bars.

It has been shown that MCAO induces major changes in the peripheral immune system, including significant spleen atrophy in male mice (Offner et al., 2006). Therefore we assessed spleen weight and immune cell composition in non-pregnant/non-postpartum mice with and without intranasal oxytocin treatment and in nursing postpartum mice after MCAO (experimental overview illustrated in Figure 3A). Figure 4A demonstrates that oxytocin induced a moderate 1.4-fold increase in spleen weight in females, while nursing resulted in a stronger 2.2-fold increase in spleen weight compared to control sham. MCAO caused small but significant decreases in spleen weights in all groups (17% decrease in control, 26% in oxytocin treated, and 14% in nursing mice). We then studied male Swiss Webster mice and found modest decreases in spleen weight following MCAO, with no significant effect of oxytocin treatment on spleen weight (Supplementary Material).

**FIGURE 5:**
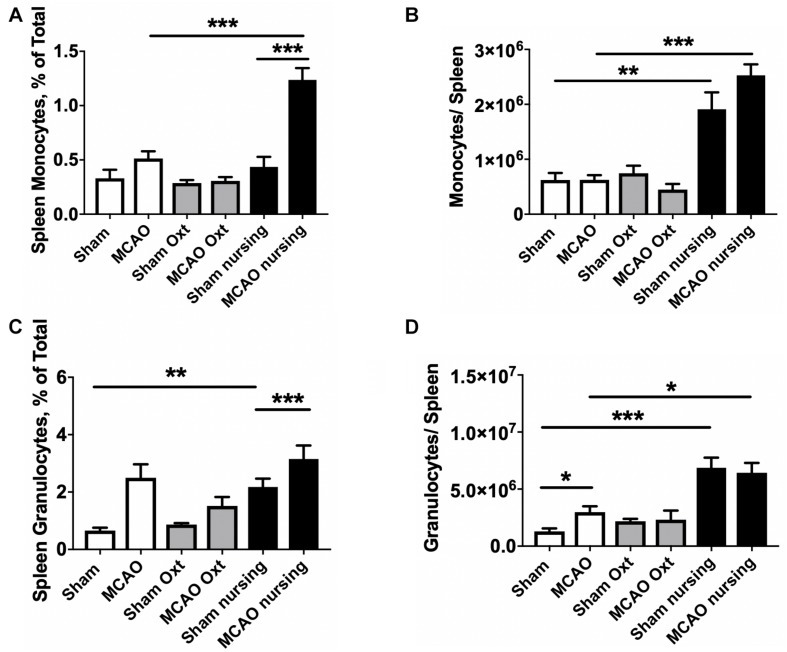
Nursing- and oxytocin (Oxt)-associated changes in spleen myeloid cells. Monocyte frequency **(A)** and total counts **(B)** demonstrated significant increase in nursing animals. Granulocyte frequencies **(C)** and total counts **(D)** were also increased in nursing animals. *N* = 4, ^∗∗∗^*p* < 0.001, ^∗∗^*p* < 0.01, and ^*^p < 0.05 for comparisons indicated by horizontal bars.

Analyses of spleen cell suspensions by flow cytometry indicate that nursing was associated with a significant 1.7-fold increase in total spleen leukocytes, while oxytocin treatment resulted in only a non-significant increase in non-ischemic animals. Ischemia caused a significant 37% decrease in total spleen leukocyte numbers in control animals. Significant MCAO-associated decreases in total spleen leukocytes were also observed in oxytocin-treated and nursing mice (45 and 36%, respectively, Figure 4B). We observed nursing-associated changes in spleen myeloid cells, monocytes and granulocytes. Figures 4C,D demonstrate sequential gating strategies used to analyze myeloid cells in the spleen and in the brain, respectively. Analysis of monocyte frequency and total counts showed a markedly increased frequency in nursing post-MCAO animals, compared to all other groups (Figure 5A). This translated to significantly higher spleen monocyte counts in the nursing mice, without significant differences between non-ischemic and post-MCAO animals, due to the differences in overall leukocyte count (Figure 5B). Granulocyte frequency was significantly increased in non-ischemic nursing mice, compared to non-ischemic sham control (Figure 5C). The total spleen granulocyte numbers were significantly increased in both ischemic and post-MCAO nursing mice, compared to corresponding sham controls (Figure 5D). However oxytocin supplementation did not recapitulate the effects observed with nursing (Figures 5A–D).

**FIGURE 6:**
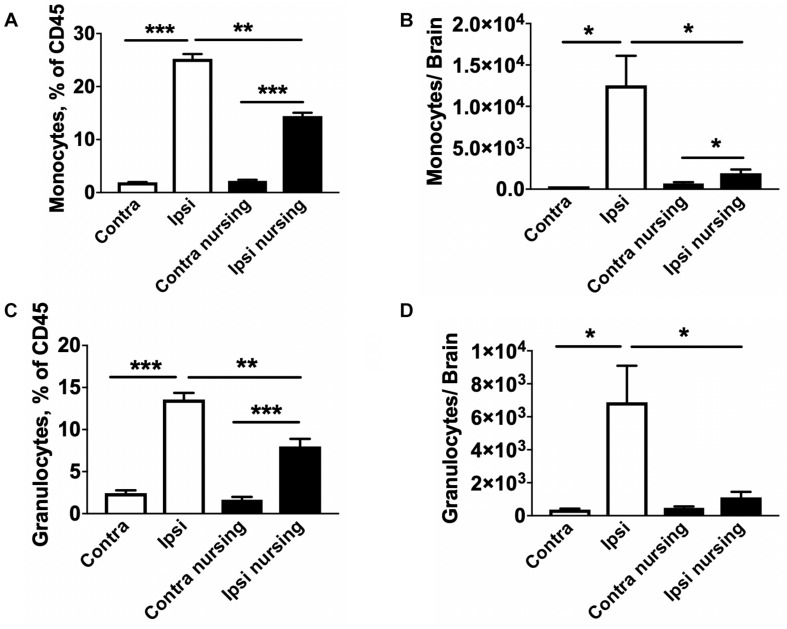
MCAO-induced brain migration of monocytes and granulocytes is reduced in nursing mice. In non-nursing mice MCAO promoted strong migration of monocytes **(A,B)** and granulocytes **(C,D)** into the ipsilateral ischemic hemispheres (Ipsi) compared to the non-ischemic contralateral hemispheres (Contra). This migration was significantly attenuated in nursing mice. *N* = 4, ^*^p < 0.05, ^∗∗^*p* < 0.01, and ^∗∗∗^*p* < 0.001 for comparisons indicated by horizontal bars.

Lymphocyte populations in the spleen were also analyzed. We observed a significant 2.1-fold increase in total regulatory T cells (Treg) numbers in spleens of post-MCAO nursing animals compared to control ischemic animals, while only a non-significant 1.4-fold increase was observed in oxytocin-treated mice. Results of FACS studies for different leukocyte types, frequency and total counts, are included in Supplementary Material. Because leukocyte migration into the brain develops over time and peaks around 3 days post-stroke (Jin et al., 2010), we performed FACS studies of brain immune cells at this time point. MCAO induced a strong 3.3-fold increase in total leukocyte numbers in the stroke affected (ipsilateral) brain hemisphere compared to the contralateral hemisphere in control mice (33583 vs. 15077 total leukocytes in ipsilateral vs. contralateral hemispheres). This MCAO-associated increase was significantly attenuated in nursing mice (13052 total leukocytes per hemisphere) only reaching levels comparable to the non-ischemic hemispheres of controls. This was paralleled by significant increases in frequencies (Figure 6A) and total cell numbers of monocytes (Figure 6B) and granulocytes (Figures 6C,D) in the ipsilateral hemispheres of MCAO animals. Together our observations demonstrate that nursing significantly attenuated the MCAO-induced increase of immune cell types that are considered central to the development of ischemic injury (Shi and Pamer, 2011).

## Discussion

Oxytocin is essential for lactation and postpartum maternal behavior (Gimpl and Fahrenholz, 2001). Recent studies report significant neuroimmunological changes in postpartum animals (Haim et al., 2017; Sherer et al., 2017), and immune responses are crucial to stroke outcome (Stoll et al., 1998; Schwab et al., 2001; Iadecola and Anrather, 2011). Our results show for the first time that nursing animals have markedly smaller infarct volumes and improved neurological outcome following MCAO injury. To compare the protective effects of nursing and oxytocin treatment we administered oxytocin intranasally and achieved brain oxytocin levels comparable to those in nursing animals. We observed reduced infarct volume and improved neurological outcome in oxytocin-treated female mice. These results support related observations in male mice whereby oxytocin treatment improved stroke outcome after cerebral ischemia when caged alone, but provided no protection to group-housed male mice that had higher brain oxytocin levels (Karelina et al., 2011).

Oxytocin inhibited LPS-induced inflammation in microglial cells and attenuated microglial activation associated with LPS treatment and maternal separation *in vivo* (Yuan et al., 2016; Amini-Khoei et al., 2017). Stroke induces significant inflammation in the brain (Choe et al., 2011; Vogelgesang et al., 2014); however, to the best of our knowledge, the effects of oxytocin on post-stroke inflammation have not been studied. Our studies indicate that the levels of two major pro-inflammatory cytokines associated with stroke injury, TNF-α and IL-6 (Campbell et al., 1993; Rothwell and Relton, 1993; Meistrell et al., 1997; Lavine et al., 1998), were elevated following MCAO injury. Independently, both oxytocin treatment and nursing significantly attenuated this pro-inflammatory cytokine release. The decrease in pro-inflammatory cytokine levels in nursing mice was accompanied by reduced brain migration of blood leukocytes, particularly monocytes and granulocytes. These cell types have been shown to be central to the development of post-ischemic brain inflammation and damage, especially due to pro-inflammatory cytokine generation (Shi and Pamer, 2011). Thus one major mechanism of protection is likely reduced migration of monocytes and granulocytes to the brain.

Peripheral immune responses are activated by stroke and interact with the development of brain damage. The spleen is a key lymphatic organ and a major reservoir of blood cells that come into the circulation and brain following brain injury (Mebius and Kraal, 2005). Therefore, splenic responses after stroke have gained attention (Seifert and Offner, 2018). It is well established that the spleen shrinks in animal stroke models (Offner et al., 2006; Ajmo et al., 2009). Spleen shrinkage is associated with early release of splenocytes and splenocyte apoptosis (Liu et al., 2015). Our studies demonstrate that nursing is associated with a marked increase in spleen size, and reduced MCAO-associated spleen atrophy. Spleen responses have been shown to correlate inversely with infarct volume (Vendrame et al., 2006), and, in turn, influence the development of ischemic brain injury (Liu et al., 2015). Prior studies have shown that splenectomy, preceding or immediately after stroke, or spleen irradiation is protective (Dotson et al., 2014; Chauhan et al., 2018). To the best of our knowledge this study describes the first observations of nursing-associated reduced spleen atrophy in post-MCAO animals, with associated improvements in stroke outcomes. In the present study we also observed that nursing independently promoted significant increases in spleen weight and total leukocyte numbers prior to injury. Importantly, nursing resulted in higher numbers of spleen monocytes and granulocytes, and this increase was retained after stroke induction. Both monocytes and granulocytes have been shown to contribute to post-ischemic brain damage and inflammation, and their increased numbers in the spleen apparently inversely correlate with the observed decrease in those cell types in post-ischemic brain (Shi and Pamer, 2011). Notably, in the present study we observed that oxytocin treatment alone failed to significantly reduce the MCAO-associated spleen atrophy.

It has been demonstrated that pro-inflammatory cytokine production induced by LPS stimulation is attenuated during pregnancy and in the postpartum period (Sherer et al., 2017). Whether lactation or oxytocin treatment leads to reduced peripheral immunosuppression remains to be determined. Post- and peripartum changes in baseline microglial density, and brain levels of interleukins 6 and 10, have been previously described in rat brains (Haim et al., 2017). Notably, the brain immune response to stress (forced swim test) is also altered in pregnant versus non-pregnant females. Most relevant to the present study, Ritzel et al. (2017) recently described reduced baseline microglial activity in multiparous female rats compared with nulliparous rats. This translated to reduced inflammatory activation after experimental stroke, reduced injury and faster recovery. Because infection is a major factor in post-stroke mortality this will be an important future direction to pursue.

Recent studies also suggest that Treg cells, a subset of T lymphocytes, are beneficial for stroke outcome (Liesz et al., 2009; Li et al., 2013). While significant Treg brain migration is generally observed at later time points than studied here, it has been shown that Treg depletion promotes increased levels of pro-inflammatory cytokines in the blood of post-ischemic animals within hours after MCAO (Liesz et al., 2009). In the present study we observed that nursing was associated with increased total spleen Treg cells, suggesting that this mechanism may also contribute to the observed protection, though future studies at later time points will be required.

## Summary

This study is the first to assess the effects of nursing and exogenous oxytocin treatment in stroke outcomes in female mice. Our study demonstrates for the first time strong nursing-associated neuroprotection against experimental stroke, along with observed oxytocin-associated anti-inflammatory mechanisms. Spleen and brain monocyte numbers were also increased with nursing, whereas in nursing mouse brains monocyte number and fraction were both decreased following MCAO. These studies suggest that: (1) intranasal oxytocin may be a novel neuroimmunological approach to reduce injury from stroke in females; and, (2) that nursing confers protection against neuroinflammatory changes and resultant stroke injury. One limitation of the present study is that the effect of oxytocin replacement therapy on stroke outcomes and neuroimmune modulation was not assessed in non-nursing postpartum females. As these studies did not include a non-nursing group for all comparisons, changes in postpartum females following MCAO cannot necessarily be attributed to nursing *per se*, as other features associated with pup presence/absence could also underlie our observations. Litter size may also have an effect on postpartum stroke outcomes, as nulliparity versus multiparity have bene shown to determine stroke outcomes (Ritzel et al., 2017), however this was not controlled for in this study. A final limitation is that we did not identify the cell-type specific source of brain cytokine modulation associated with oxytocin treatment. Further studies incorporating cell-type specific mechanistic studies will be required to develop a more comprehensive understanding of the regulatory pathways responsible for lactation-associated neuroprotection, to advance therapeutic applications for this specific, at-risk population.

## Data Availability

The datasets generated for this study are available on request to the corresponding author.

## Ethics Statement

This study was carried out in accordance with the recommendations of the National Institutes of Health guidelines for the use of experimental animals. The protocol was approved by the Stanford Animal Care and Use Committee.

## Author Contributions

CS contributed to study design, data analysis, and manuscript preparation. LX performed the animal surgeries. LV performed the ELISA experiments. MA-H performed the FACS experiments. OA performed the blinded treatments, tissue collection, and histological analyses. JI contributed to study design and data analysis. RG contributed to study design, data analysis, and manuscript preparation.

## Conflict of Interest Statement

The authors declare that the research was conducted in the absence of any commercial or financial relationships that could be construed as a potential conflict of interest.
