# Supplementary material for: Nursing Markedly Protects Postpartum Mice From Stroke: Associated Central and Peripheral Neuroimmune Changes and a Role for Oxytocin
Source: Front Neurosci. 2019 Jul 8;13:609. doi: 10.3389/fnins.2019.00609 (PMC6637858; doi:10.3389/fnins.2019.00609)
Supplement: Supplementary file 1 [file Data_Sheet_1.PDF]

Supplemental Material

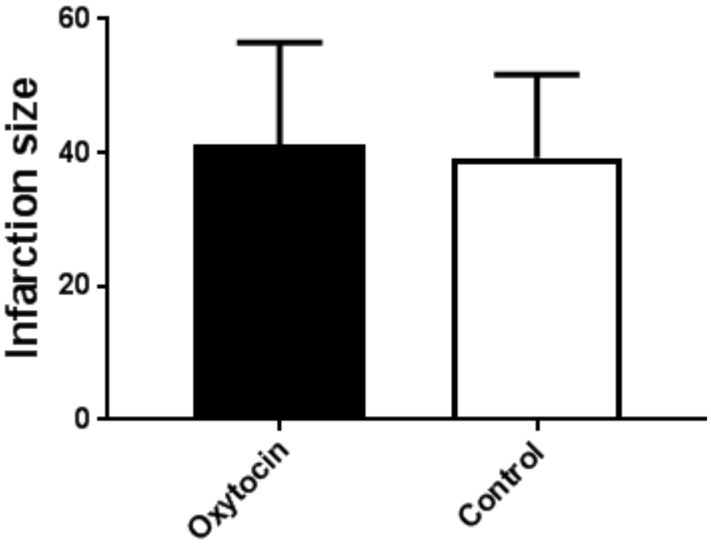

N=12 N=11

Supplemental Fig 1 The effect of oxytocin treatment on post-MCAO outcome in group housed male mice. Intranasal oxytocin treatment for 3 days resulted in no significant changes in the infarction size

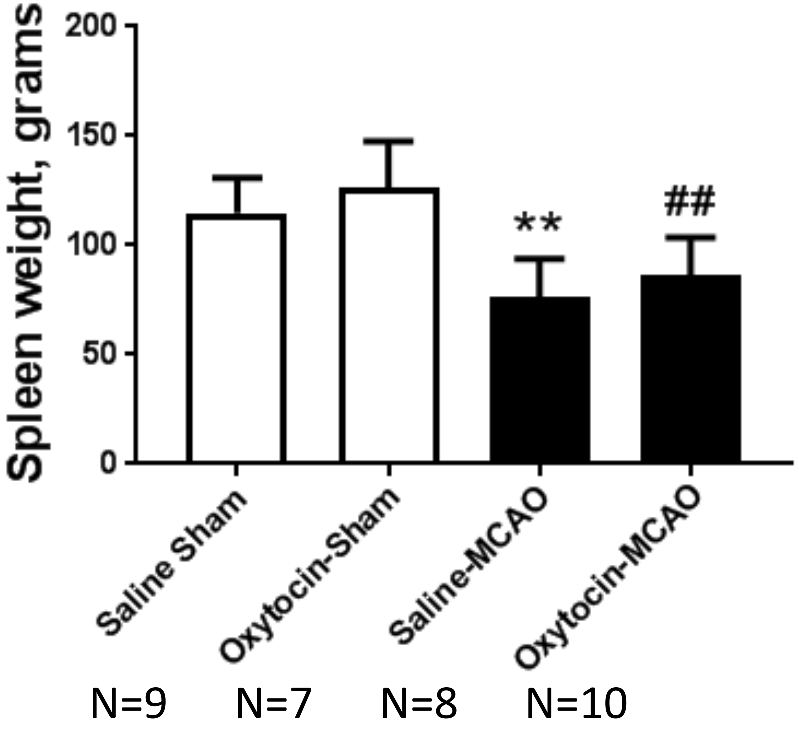

Supplemental Fig 2 Changes in spleen weight with or without oxytocin treatment in non-ischemic and post-MCAO male mice. Non-ischemic animals demonstrated significantly higher spleen weights compared to MCAO animals (**$p<0.01$ compared to saline sham, ##$p<0.01$ compared to oxytocin sham).

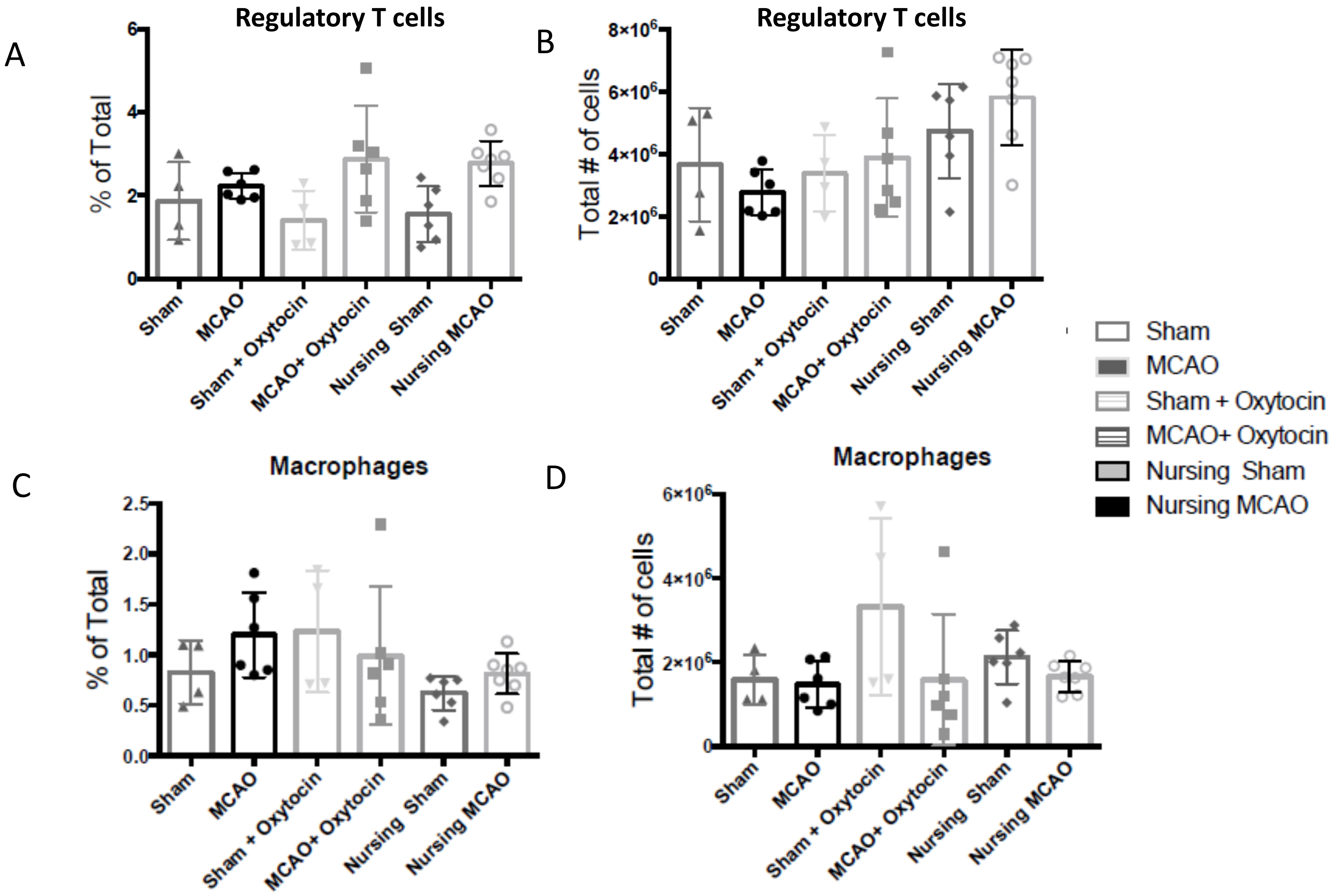

Supplemental Fig 3 Lactation and oxytocin treatment associated changes in spleen regulatory T cell frequency (A) and total counts (B) and macrophage frequency (C) and total counts (D).

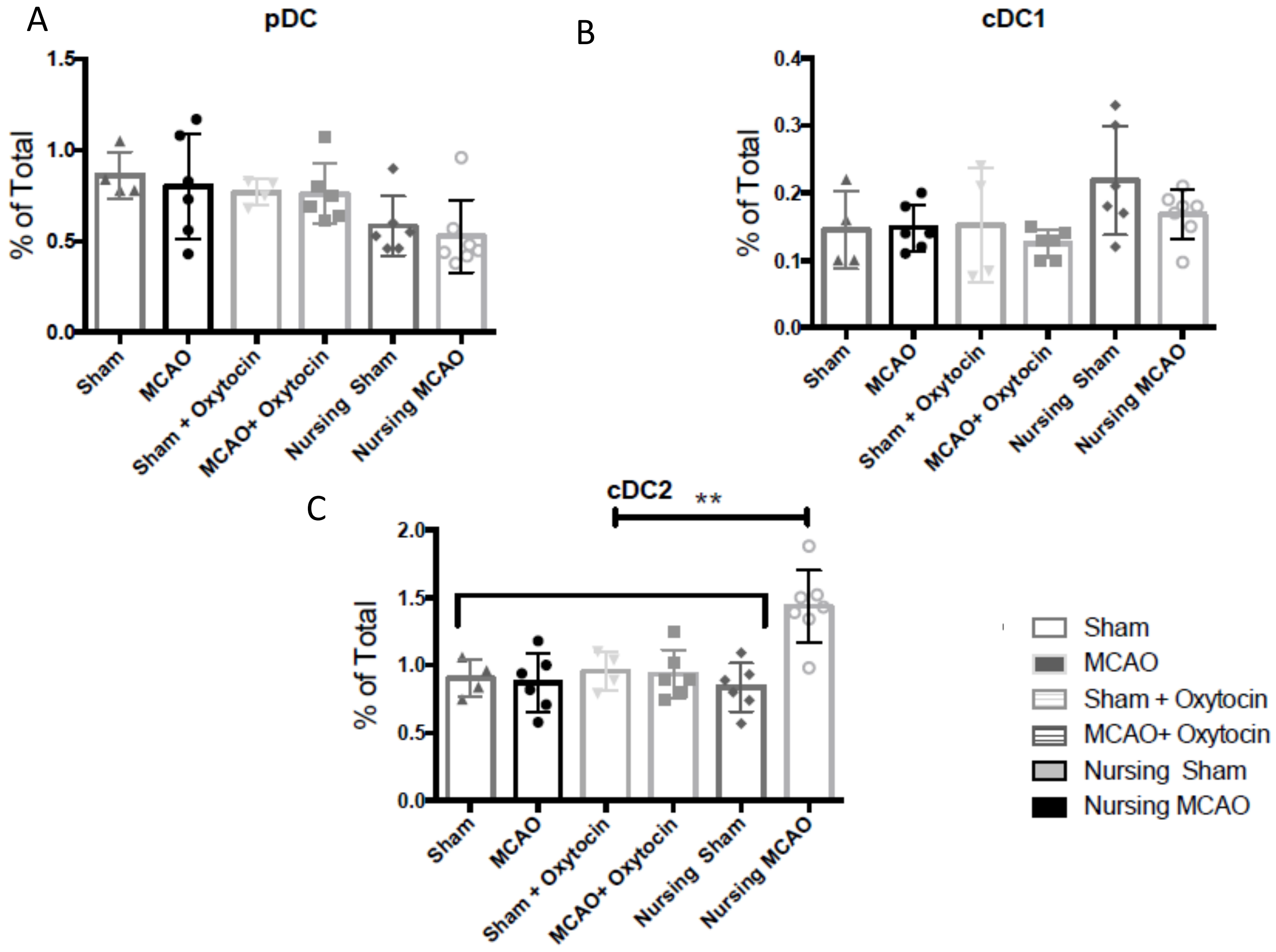

Supplemental Fig 4 Lactation and oxytocin treatment associated changes in frequency of spleen plasmacytoid DC (A), cDC1 (B), DC2 (C) .

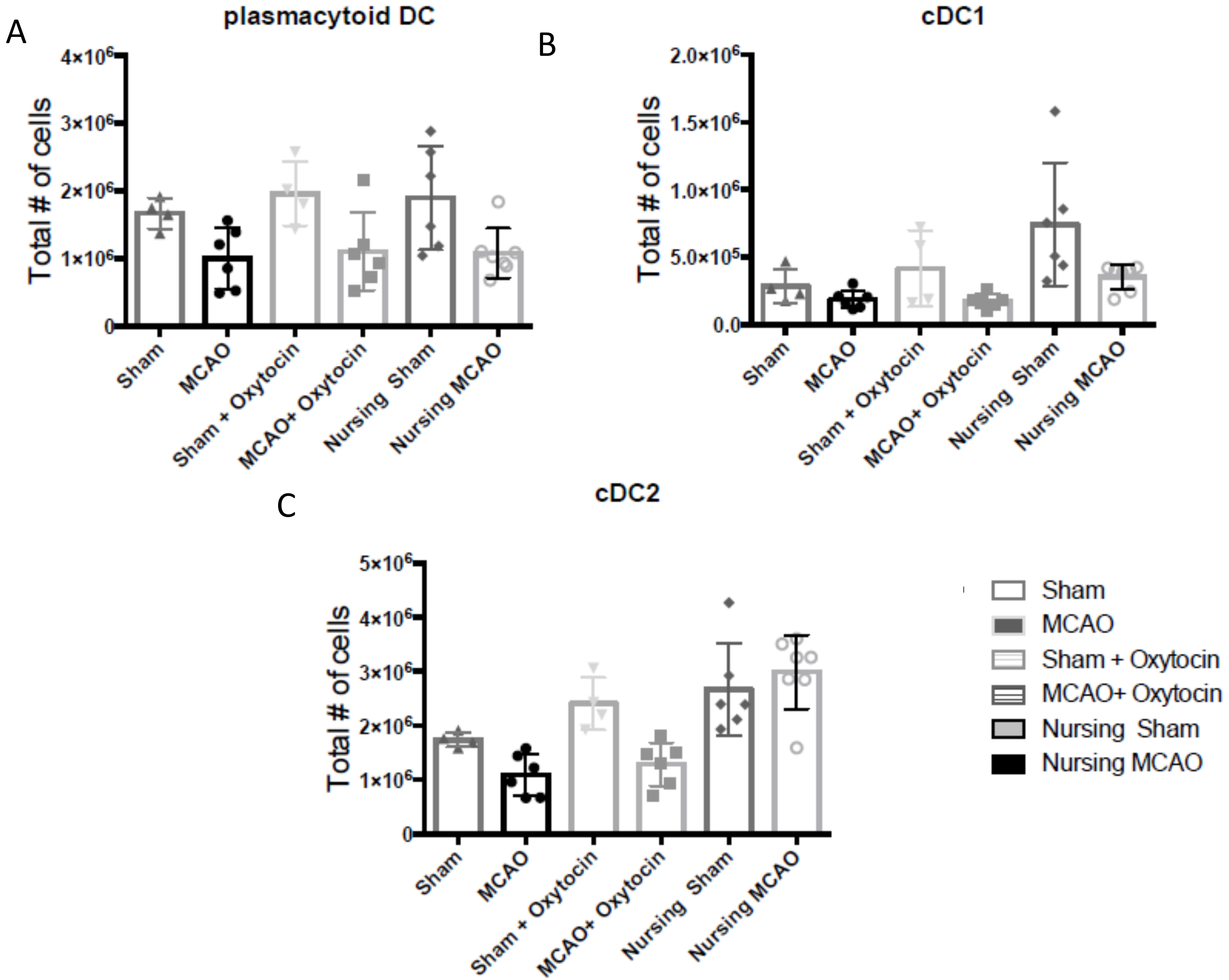

Supplemental Fig 5 Lactation and oxytocin treatment associated changes in total counts of spleen plasmacytoid DC cells (A), cDC1 cells (B), DC2 cells (C) .

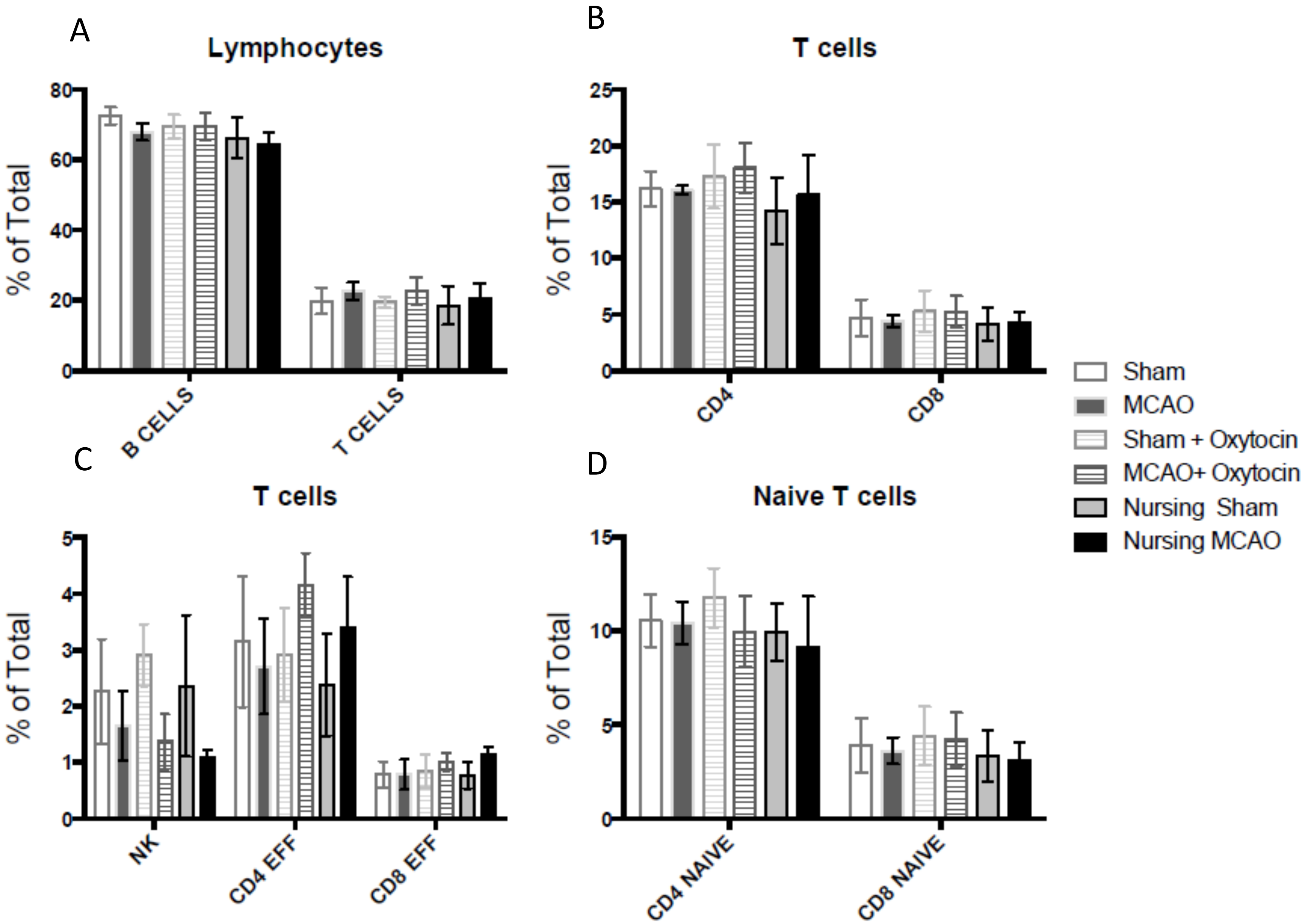

Supplemental Fig 6 Lactation and oxytocin treatment associated changes in frequency of spleen lymphocytes (A), and different subgroups of T cells (B-D).

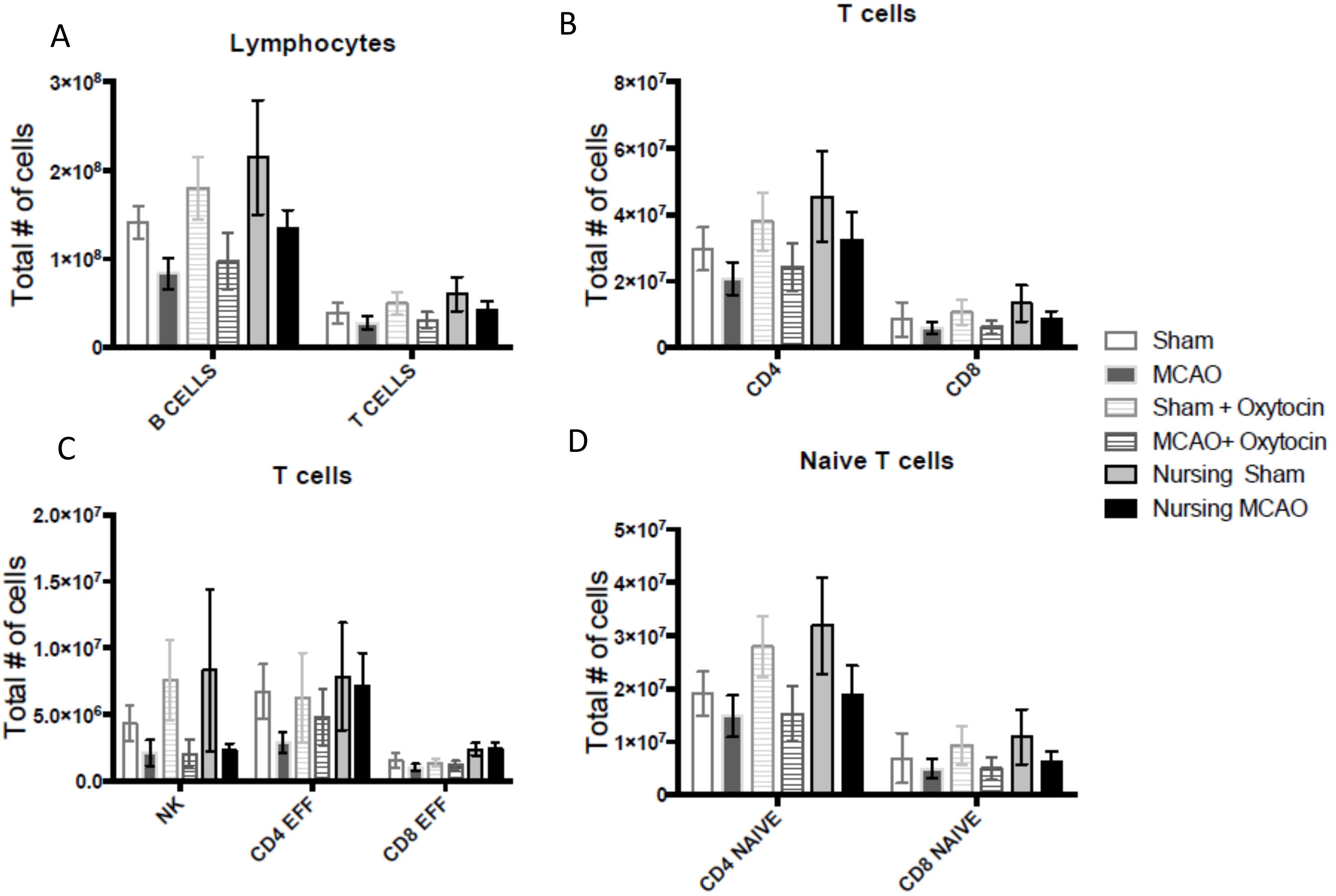

Supplemental Fig 7 Lactation and oxytocin treatment associated changes in total counts of spleen lymphocytes (A), and different subgroups of T cells (B-D).
